# Bipedicular percutaneous kyphoplasty versus unipedicular percutaneous kyphoplasty in the treatment of asymmetric osteoporotic vertebral compression fractures: a case control study

**DOI:** 10.1186/s12893-023-02180-7

**Published:** 2023-09-19

**Authors:** Lei Liu, Feifei Song, Jie Shang, Jianwei Zhang, Chao Ma, Guangpu Liu, Meng Han

**Affiliations:** 1 Department of Spinal surgery, Xuzhou Central Hospital, 199 Jiefang South Road, Xuzhou, Jiangsu China; 2 Department of Spinal Surgery, Xuzhou Clinical School of Xuzhou Medical University, Xuzhou, Jiangsu China

**Keywords:** Osteoporosis, Vertebral compression fracture, Puncture path, Percutaneous kyphoplasty, Asymmetric compression fracture

## Abstract

**Background:**

Bipedicular/unipedicular percutaneous kyphoplasty are common treatments for OVCF, and there are no studies to show which is more beneficial for AVCF. The purpose of this study was to investigate the clinical efficacy of BPKP or UPKP in the treatment of AVCF.

**Methods:**

The clinical data of AVCF patients treated by PKP were retrospectively analyzed. They were divided into two groups according to the surgical approach. General demographic data, perioperative complications, and general information related to surgery were recorded for both groups. The preoperative and postoperative vertebral height difference, vertebral local Cobb angle, lumbar pain VAS score and lumbar JOA score were counted for both groups. The above data were compared preoperatively, postoperatively and between the two groups.

**Results:**

25 patients with AVCF were successfully included and all were followed up for at least 12 months, with no complications during the follow-up period. 10 patients in the BPKP group and 15 patients in the UPKP group, with no statistically significant differences in general information between the two groups. The VAS scores of patients in the BPKP group were lower than those in the UPKP group at 12 months after surgery, and the differences were statistically significant, and there were no statistically significant differences between the two groups at other follow-up time points. In the BPKP group, 80% of patients had symmetrical and more homogeneous bone cement dispersion. 50% of patients in the UPKP group had a lateral distribution of bone cement and uneven bone cement distribution, and the difference in bone cement distribution between the two groups was statistically significant.

**Conclusion:**

For the treatment of AVCF, the clinical efficacy of both surgical approaches is basically the same. The distribution of cement is more symmetrical and uniformly diffused in the BPKP group, and the clinical efficacy VAS score is lower in the long-term follow-up. Bipedicular percutaneous kyphoplasty is recommended for the treatment of AVCF.

**The ethical review batch number:**

XZXY-LJ-20161208-047.

## Background

Osteoporotic vertebral compression fracture (OVCF) is a common kind of fracture that endangers the health of the elderly. Percutaneous kyphoplasty (PKP) is a widely applied procedure for OVCF, with the advantages of minimal trauma, minimal bleeding, rapid recovery, and high safety. It is generally considered safe and effective and is therefore recommended as the method of choice for OVCF. Different approaches in PKP have their own merits and shortcomings. While the unilateral approach has the advantages of short operative time and less fluoroscopy, it has the disadvantages of arch fracture and uneven cement distribution. On the other hand, the bilateral approach has the advantages of more cement injection and uniform cement distribution, and the disadvantages of longer operative time, more fluoroscopy, and cement separation. In some patients, as the pressure on the fractured vertebrae is not completely uniform and symmetrical, the fractured vertebrae may produce asymmetrical compression changes. The local deformity may lead to complications such as residual pain, local scoliosis deformity, and vertebral recompression fracture in the distant future. Therefore, asymmetric osteoporotic vertebral compression fractures (AVCF) require not only relief of lumbar pain and restoration of vertebral height but also correction of local scoliosis deformity. Heretofore, no relevant studies have shown which approach is more suitable for the treatment of AVCF. We retrospectively analyzed the clinical data of patients who attended our hospital for PKP for AVCF from January 2020 to December 2021 to determine through a controlled study which surgical approach is more suitable for the treatment of AVCF.

This study was approved by the ethics committee of our hospital for consent.

## Inclusion and exclusion criteria

Inclusion criteria: ①_clear diagnosis of OVCF without symptoms of neurological injury, number of fractured vertebrae 1–2. ②_no external violence, ③_exclusion of pathological fractures due to infection or tumor, ④_unilateral or bilateral transcatheter root approach PKP surgery, ⑤_fractured vertebra type AVCF, and ⑥_follow-up time of at least 12 months.

Exclusion criteria: ①_follow-up time is less than 12 months, ②_the number of fractured vertebrae is greater than 2, ③_vertebral fracture is a pathological fracture caused by tumor or infection or burst fracture caused by violence, or fracture causes related neurological injury symptoms, ④_the surgical method is extra-arch root approach PKP or open surgery.

## Methods

Patients who underwent PKP surgery for OVCF at our institution from January 2020 to December 2021 were retrospectively selected, and 312 patients were found to be eligible for the study. Finally, 25 patients with AVCF were included in the final study in accordance with the inclusion and exclusion criteria. The general data of the patients were studied, including general information such as gender, age, comorbidities, and days of hospitalization. The patients’ surgical procedure, fractured vertebrae, operative time, number of fluoroscopies, cement distribution pattern, complications, and cement injection volume were recorded. The pre-and postoperative height difference between the two sides, postoperative height recovery rate, pre- and postoperative local Cobb angle of the injured vertebrae, and local Cobb recovery rate were measured and recorded on PACS based on the patient’s imaging data. The preoperative, three-day postoperative, and one-year postoperative visual analog scale (VAS) scores and lumbar Japanese Orthopaedic Association (JOA) scores were counted. According to the different surgical methods of the patients, they were divided into the bilateral pedicle puncture group (BPKP group) and the unilateral pedicle puncture group (UPKP group). The clinical data between the two groups were statistically analyzed.

A fracture was classified as an AVCF: when the vertebral body height difference was found at the position of the medial edge of the pedicle projection on both sides of the patient’s fractured vertebral body on the anteriorposterior orthopantomograph. Only one vertebral edge was compressed, or both vertebral edges appeared to be compressed but to different degrees.

**Fig. 1:**
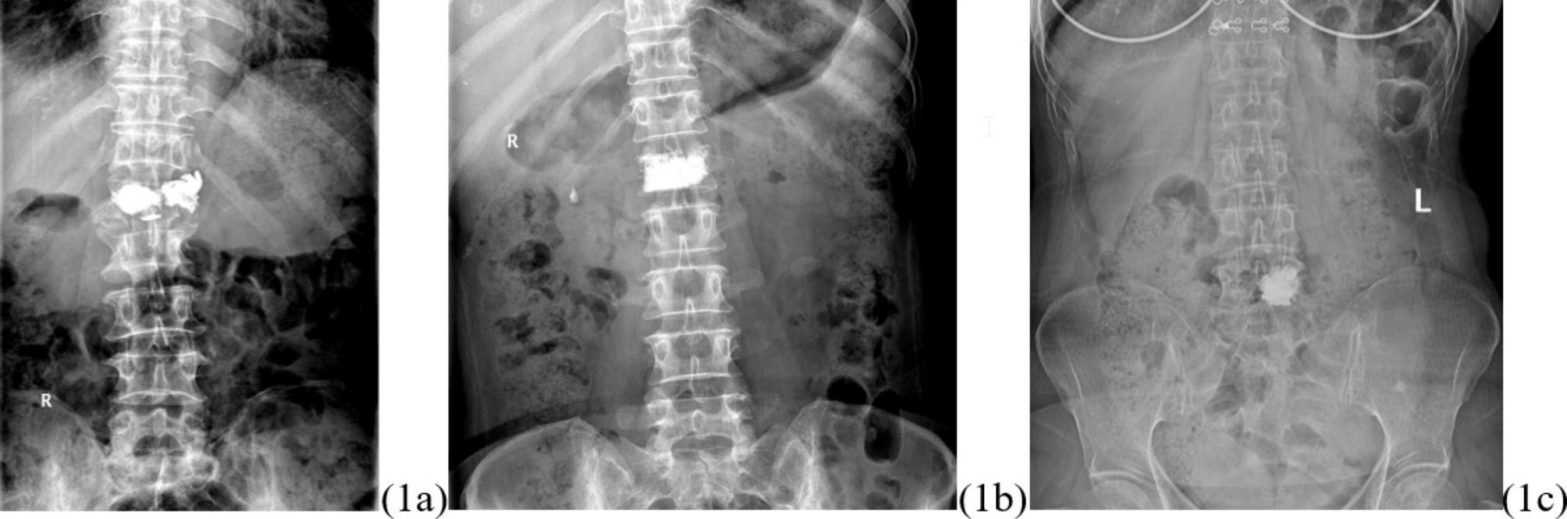
Distribution pattern of bone cement: symmetrical block distribution **(1a)**, symmetrical spongy distribution **(1b)**, asymmetrical block distribution (off-side) **(1c)**

The cement distribution pattern was symmetrical or asymmetrical on one side, clumped together in a mass, or evenly dispersed like a sponge. (Fig. 1)

The local Cobb angle is the angle formed by the horizontal line where the upper and lower endplates of the fractured vertebral body are located or their extensions.

## Surgical methods

Each patient was placed in the prone position, and the C-arm machine was fluoroscoped and adjusted until the standard orthogonal and lateral images of the vertebral body were displayed. After disinfection, a surgical sheet was placed and local infiltration anesthesia was administered. An incision of approximately 0.5 cm in length was made approximately 2 cm above the spinous process of the fractured vertebra. The puncture needle was inserted, and the tip was adjusted under fluoroscopy to be located at the lateral superior edge of the arch root shadow, and the puncture needle was inserted into the arch root, with the tip located at the medial edge of the arch root shadow in the frontal view and at the posterior edge of the vertebral body in the lateral view. A long guide needle was placed, and fluoroscopy displayed a good position, and a working trocar was inserted. Patients with bilateral access required the same approach to place a working trocar on the other side of the pedicle.

The balloon was placed and opened, and after fluoroscopy displayed no significant increase in the vertebral body height, the balloon was removed. Patients with bilateral access completed the expansion on the other side. Bone cement was prepared and awaited to be pushed in through the minimally invasive tool at the later stages of the draw. Fluoroscopy revealed no leakage of bone cement to the posterior aspect of the vertebral body. After waiting for the bone cement to be set, it was again mixed and injected in a certain amount, and fluoroscopy showed the position of the bone cement. Bilateral access patients underwent bilateral access for bone cement injection, followed by disinfection and dressing of the incision. Patients were checked for good voluntary movement of both lower extremities, and the procedure was completed.

## Statistical methods

Data were statistically analyzed using SPSS 20.0 software. The measurement data conforming to normal distribution were expressed as mean ± standard deviation (x ± s), and the count data were expressed as n. Either the independent samples t-test or chi-square test was used for comparison between groups, and repeated-measures data ANOVA was used for comparison of observed indicators at different time points. The independent simples t-test was used for inter-group comparison of measurement data, and the chi-square test was used for inter-group comparison of counting data. Differences were considered statistically significant at p < 0.05.

## Results

Clinical data were obtained from 312 patients with OVCF, and 25 patients with AVCF were finally included in this study, with an incidence of AVCF of 8.01% (25/312). The patient population included seven males and 18 females, aged 59–91 years, with a mean age of 74.560 ± 8.903 years. The distribution of fractured vertebrae was T10_L5 vertebrae. The hospital stay was 3_9 days, with an average of 5.400 ± 1.826 days, and the operative time was 30_60 min, with an average of 45.200 ± 9.734 min. Seven cases had hypertension, four cases had diabetes mellitus, three cases had kyphosis, four cases had lumbar spinal stenosis, and one case had cerebral infarction (Table 1).

**Table 1:** General information of patients

		All	BPKP Group	UPKP group	t/X^2^	p
n		25	10	15		
Gender	Male/femal	7/18	2/8	5/10	0.529	0.467
Age	(year)	74.560 ± 8.903	79.800 ± 5.453	71.067 ± 9.169	0.129	0.113
Associated disease					2.475	0.65
	Hypertension	7	2	5		
	Diabetes	4	1	3		
	Kyphosis	3	2	1		
	Lumbar spinal canal stenosis	4	2	2		
	History of cerebral infarction	1	0	1		
Fractured vertebrae					8.993	0.253
	T10	1	1	0		
	T11	1	0	1		
	T12	5	1	4		
	L1	8	3	5		
	L2	3	2	1		
	L3	2	2	0		
	L4	3	0	3		
	L5	2	1	1		

The differences in general information between the two groups were not statistically significant (Table 1).

In the BPKP group, the fractured vertebrae were located in the T10_L5 vertebrae except T11 and L4, and cement leakage occurred in two patients. In the UPKP group, the fractured vertebrae were located in the T11_L5 vertebrae, except L3, and cement leakage occurred in seven patients. No statistically significant difference was found in the mean number of hospital days, mean operative time, mean number of fluoroscopic views, and mean amount of bone cement injected between the two groups. Eight patients in the BPKP group had a symmetrical distribution of bone cement, and five patients in the UPKP group had a symmetrical distribution, and the difference in the distribution of bone cement between the two groups was statistically significant (p = 0.022; Table 2).

**Table 2:** General information of patients’ surgery

		All	BPKP Group	UPKP group	t/X^2^	p
n		25	10	15		
Bone cement distribution					5.235	0.022
	Symmetric	13	8	5		
	Asymmetric	12	2	10		
Days of hospitalization	(day)	5.400 ± 1.826	5.900 ± 1.524	5.067 ± 1.981	1.124	0.273
Operation time	(min)	45.200 ± 9.734	43.500 ± 12.259	46.333 ± 7.898	0.705	0.488
Number of fluoroscopy		64.560 ± 14.748	65.500 ± 13.914	63.933 ± 15.727	0.255	0.801
Bone cement injection volume	(ml)	8.160 ± 1.772	7.8 ± 1.398	8.400 ± 1.993	0.824	0.148
Bone cement leakage		9	2	7	2.852	0.174

Patients in both groups had improved VAS scores and lumbar JOA score scores compared with the scores in the preoperative period. At the final follow-up only, the difference in VAS scores between the two groups was statistically significant (p = 0.005). The differences in pre- and postoperative VAS scores and JOA scores between the two groups at the remaining time points were not statistically significant (Table 3).

**Table 3:** Preoperative and postoperative VAS scores and JOA scores

	BPKP Group	UPKP group	t	p
Pre-operative VAS	7.900 ± 1.449	8.467 ± 1.457	0.955	0.35
VAS 3 days after surgery	3.900 ± 1.287	4.2 ± 1.01419	0.651	0.521
VAS 1 year after surgery	3.000 ± 1.247	4.267 ± 0.799	0.664	0.005
Pre-operative JOA	14.300 ± 2.163	14.000 ± 1.890	0.367	0.717
JOA 3 days after surgery	19.300 ± 2.111	18.600 ± 1.765	0.899	0.378
JOA 1 year after surgery	18.700 ± 1.494	19.067 ± 1.831	0.526	0.604

The mean height difference between the two sides of the vertebral body in BPKP was 5.400 ± 1.430 mm before surgery and 0.100 ± 0.316 mm after surgery, and the local Cobb angle of the vertebral body was 12.700 ± 7.500degrees before surgery and 1.100 ± 1.663degrees after surgery. In the UPKP group, the average height difference between the two sides of the vertebral body was 4.200 ± 1.474 mm before surgery and 0.333 ± 0.724 mm after surgery. The local Cobb angle of the vertebral body was 10.400 ± 7.259degrees before surgery and the average local Cobb angle was 1.000 ± 1.773degrees after surgery. No statistically significant difference was found in the above indexes between the two groups of patients (Table 4).

**Table 4:** Preoperative and postoperative differences in vertebral body height(DVH) and localized scoliosis cobb angle

	BPKP Group	UPKP group	t	p
Pre-operative DVH	5.400 ± 1.430	4.200 ± 1.474	2.018	0.055
DVH 3 days after surgery	0.100 ± 0.316	0.333 ± 0.724	0.04	0.349
DVH 1 year after surgery	98.750 ± 0.039	94.560 ± 0.121	0.026	0.304
Pre-operative cobb angle	12.700 ± 7.499	10.400 ± 7.259	0.766	0.451
cobb angle 3 days after surgery	1.100 ± 1.663	1.000 ± 1.773	0.142	0.889
cobb angle 1 year after surgery	93.3 ± 0.087	87.63 ± 0.271	0.64	0.528

## Discussion

### Different surgical options for OVCF

OVCF is becoming an increasingly common threat to the health of the elderly and can be life-threatening in severe cases. A range of treatment options are available for OVCF, such as conservative treatment, percutaneous vertebroplasty, percutaneous balloon kyphoplasty, and open surgery. PKP is a safe and effective surgical procedure for the treatment of OVCF. Previous studies have shown that this surgical approach has the advantages of less trauma, faster recovery, and higher safety [1]. Bipedicular percutaneous kyphoplasty (BPKP) is the traditional surgical approach, and a few studies have demonstrated that BPKP is an effective and safe surgical procedure [2]. Unipedicular percutaneous kyphoplasty (UPKP) is gradually being performed to reduce the operative time and the number of fluoroscopic views during surgery. Both surgical approaches ultimately improve the quality of life of patients by effectively reducing perioperative pain [3]. The mean VAS score and mean lumbar JOA score of all patients in this study improved compared with the preoperative scores and the difference was statistically significant, which is consistent with the results of previous studies, indicating that both puncture methods can achieve pain relief and improve patients’ quality of life. Liu and Tan et al. noted that compared with BPKP, UPKP has the advantages of shorter operative time, less trauma, less fluoroscopy, less bone cement dosage, and lower incidence of residual pain [4] [5]. Therefore, UPKP may be the preferred method for the treatment of OVCF [6]. However, to distribute the bone cement evenly, the puncture needle will cross the midline during UPKP, and excessive pursuit of the puncture angle may entail risks such as rupture of the arch bone wall and injury to the spinal nerve. Poor cement distribution position (on one side) may bring about complications such as postoperative collapse and re-fracture of the vertebral body on the non-operating side. The results of this study showed that although the two groups of patients received different surgical approaches, there were no statistically significant differences in the number of hospital days, operative time, number of fluoroscopies, or amount of bone cement injected into individual vertebrae between the two groups. Therefore, so far, there is no uniform standard or absolute advantage in choosing which surgical approach to perform.

### Definition and clinical features of AVCF

Since OVCF is a low-energy injury, the fracture types are mostly compression fractures and mild burst fractures [7], and most of the compressed vertebrae are symmetrical wedge-shaped changes. However, some vertebrae may have asymmetric wedge changes, that is, wedge changes on one side only or wedge changes on both sides but with different heights of vertebral body loss, which we call “asymmetric vertebral compression fracture.” In this study, we reviewed the clinical data of OVCF patients in our center for one year, and the incidence rate of AVCF was 8.01%. The wedge-shaped changes in OVCF are mainly concentrated in the anterior column, and the wedge-shaped changes in AVCF are typically concentrated on both sides of the vertebral body. The mechanism of AVCF injury may be a combination of lateral flexion compression stress and a wedge-shaped fracture on the lateral edge of the vertebral body; or a symmetrical fracture initially and a secondary asymmetrical wedge-shaped change later due to improper posture of conservative treatment. After the occurrence of AVCF, the local biomechanics of the fractured vertebral body is asymmetrically altered, requiring timely intraoperative restoration of local coronal balance. After correction of the local Cobb angle in the coronal plane, the purpose of relieving clinical symptoms is achieved by stabilizing the vertebral body, balancing local biomechanics, preventing re-collapse of the vertebral body, and preventing fracture of the adjacent segment.

### Both procedures have the same pain-relieving effect for AVCF

Pain is the most common clinical manifestation in patients with AVCF. The improvement in patients’ clinical symptoms after surgery is not related to the puncture access method but is more influenced by whether the amount of bone cement injection is sufficient and whether the bone cement is evenly distributed. Theoretically, the more bone cement is injected, the higher the biomechanical strength and the better the clinical outcome. However, some studies have demonstrated that the amount of bone cement injection and the percentage of bone cement filling in the vertebral body are not directly related to the postoperative pain relief effect [8]. After the total amount of bone cement injected reaches a certain threshold, the effect of pain relief does not increase with the amount of bone cement injected. Biomechanical studies have demonstrated that BPKP/UPKP has an effect on vertebral body strength and vertebral body height changes. However, the difference in terms of clinical effect between the two is not statistically significant [9]. The choice of the surgical approach adopted does not affect the outcome of clinical relief as long as the bone cement is evenly diffused into the vertebral body. Zhang et al. conducted a clinical study of BPKP/UPKP for OVCF, and patients whose bone cement did not cross the midline after UPKP had a higher postoperative VAS score [10]. Yan et al. also concluded that the better the uniform distribution of bone cement within the vertebral body, the better the degree of clinical symptom relief after surgery [11]. The results of our study showed that the VAS scores and lumbar JOA scores of patients in both groups improved at three days and one year after surgery compared with those before surgery, and the differences between the two groups were not statistically significant, which was consistent with the results of the above study. However, at the one-year postoperative follow-up, the difference in VAS scores between the two groups appeared to be statistically significant (p = 0.005), with patients in the UPKP group having a higher VAS score. We believe that this may be related to the distribution pattern of the bone cement.

### Both surgical options have the same effect in rectifying local deformities

**Fig. 2:**
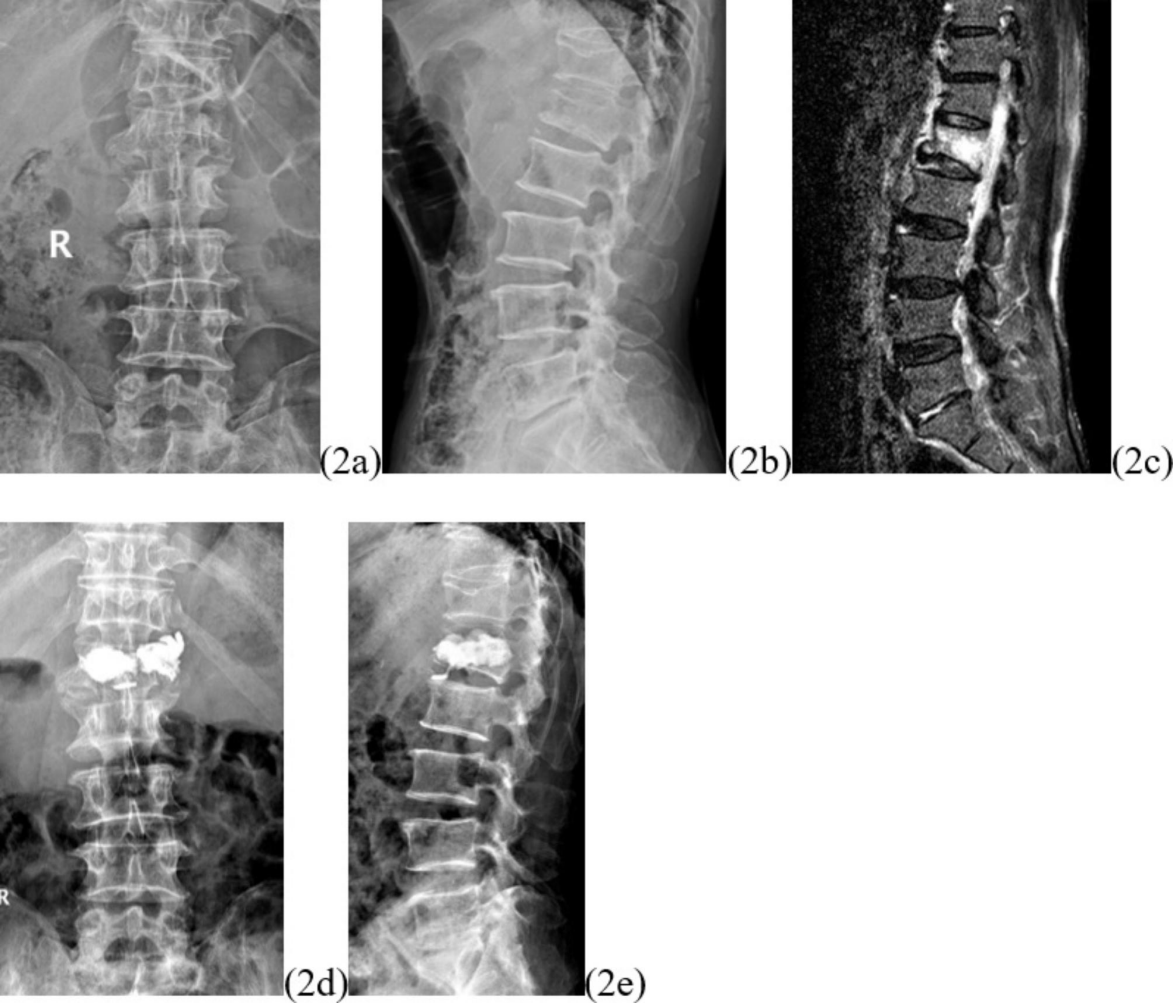
Typical case. L1 with AVCF underwent BPKP, and the postoperative bone cement showed symmetrical mass distribution. Figure 2a and b show the preoperative lumbar frontal and lateral radiographs, Fig. 2c shows the preoperative lumbar MR scan, and Fig. 2d and e show the postoperative lumbar frontal and lateral radiographs. The preoperative height difference of the injured spine was 4 mm, and the preoperative local cobb angle was 15.5°; the postoperative height difference of the injured spine was 1 mm, and the postoperative local cobb angle was 3°; the recovery rate of the height difference of the injured spine was 75%, and the recovery rate of the local cobb angle° was 80.6%

**Fig. 3:**
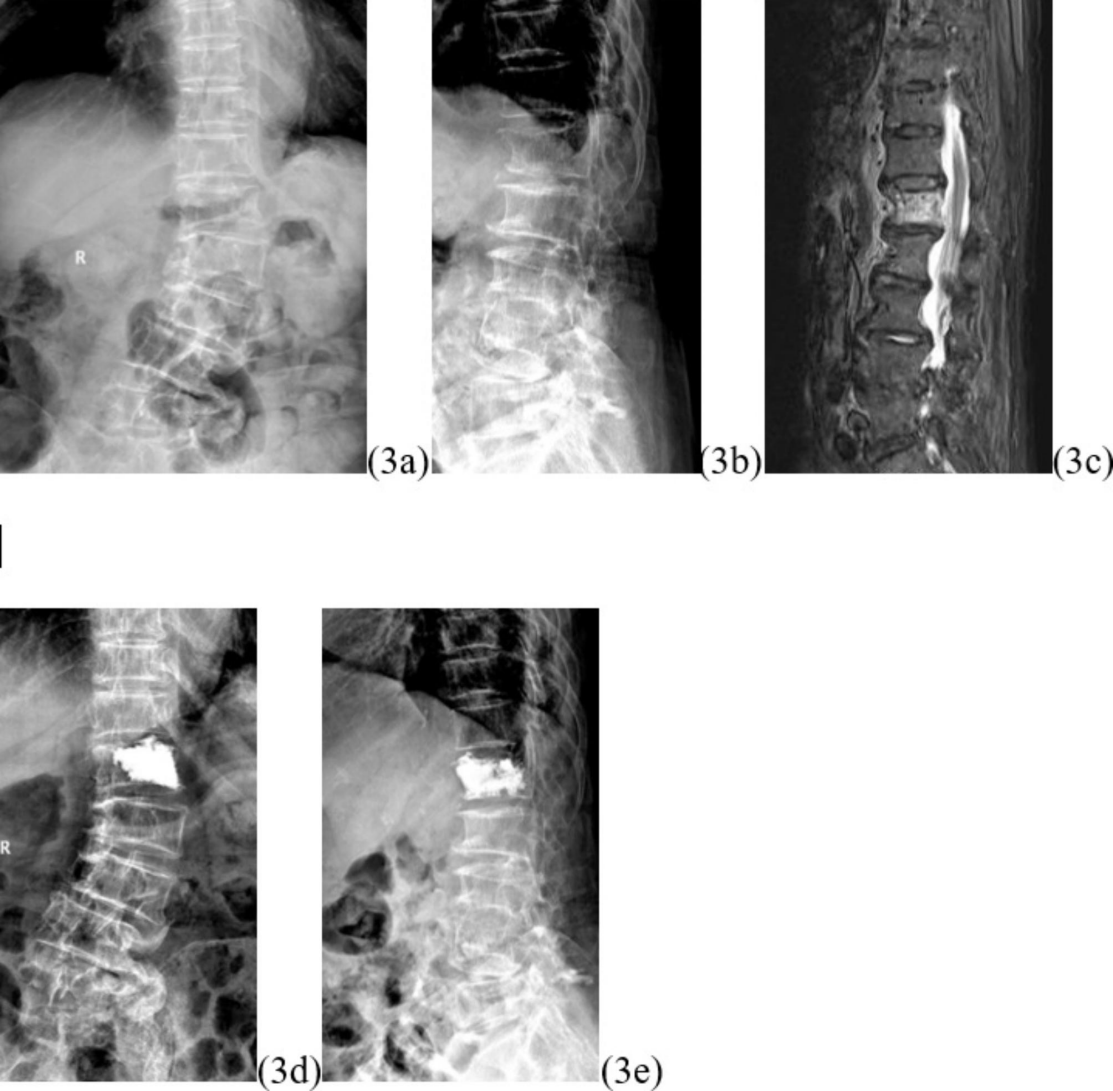
Typical case. L1 with AVCF undergoing UPKP with asymmetrical mass-like (lateralized) distribution of bone cement. Figure 3a and b show the preoperative lumbar frontal and lateral radiographs, Fig. 3c shows the preoperative lumbar MR scan, and Fig. 3d and e show the postoperative lumbar frontal and lateral radiographs.The preoperative height difference of the injured spine was 5 mm, and the preoperative local cobb angle was 16°; the postoperative height difference of the injured spine was 1 mm, and the postoperative local cobb angle was 3°; the recovery rate of the height difference of the injured spine was 80%, and the recovery rate of the local cobb angle° was 81.25%

Restoration of the height of the fractured vertebral body is one of the primary goals of the procedure. Most OVCFs are combined with localized kyphosis, and the height of the vertebral body can usually be restored to a satisfactory level by balloon expansion and prone manipulation. The restoration of vertebral body height can reduce biomechanical abnormalities brought about by the altered vertebral body height, reduce the stress on adjacent vertebrae, reduce the load on adjacent vertebrae and muscle tissue, and reduce pain of soft tissue origin [12]. So far, no consensus has been achieved concerning which among UPKP or BPKP is more effective in restoring fractured vertebral body height. The results of both Chen and Yan’s studies yielded completely opposite conclusions [13] [14]. A meta-analysis including 12 randomized controlled trials conducted by Feng et al. showed that BPKP produced better vertebral height recovery [15] Patients with AVCF have not only a kyphotic deformity, but also a combined localized scoliosis in the coronal plane. Both of these deformities should be rectified as much as possible during surgery; otherwise, re-fracture or collapse may occur on the side where vertebral height has not been restored and worsen. The results of this study showed that the differences in vertebral height difference and vertebral height recovery rates between the two groups of patients at three days and one year postoperatively were not statistically significant (Figs. 2 and 3). It suggests that both procedures are effective in maintaining fracture stability, promptly correcting scoliosis caused by asymmetric wedge changes, and effectively restoring the vertebral function of the patient.

### Same incidence of bone cement leakage

Bone cement leakage is a common complication of PKP surgery. In this study, bone cement leakage was observed in both groups and the difference was not statistically significant (p = 0.1736). However, all cement leaks did not cause nerve compression or organ damage, indicating that the leakage of bone cement was not related to the puncture access. Tan et al. conducted a meta-analysis and concluded that UPKP had a lower risk of bone cement leakage than BPKP [5]. Similar results were reported in a prospective comparative study conducted by Yan et al. [14]. The studies published by Feng and Zhang et al. however, found that bone cement leakage was not related to the puncture method [15] [16]. We believe that bone cement leakage is influenced by fracture line morphology, degree of vertebral compression, and bone density of the fractured vertebra, whether it is a fresh fracture, bone cement viscosity, and timing of bone cement injection, and that the puncture method does directly affect on bone cement leakage.

### Different surgical approaches affect the distribution of bone cement and the long-term outcome of the operation

In this study, differences were found in the symmetry of bone cement distribution between the two groups (p = 0.022). The BPKP group showed a symmetrical distribution of bone cement with homogeneous spongy distribution (2/10) or symmetrical mass-like distribution (8/10) (Fig. 2). Patients in the UPKP group showed homogeneous spongy distribution (10/115) (Fig. 3) or symmetrical mass-like distribution (5/15), and the differences between the two groups were statistically significant. Whether the bone cement is symmetrically and uniformly distributed has a great impact on the biomechanical recovery of the fractured vertebral body and plays pivotal role in maintaining vertebral body height, correcting local lateral kyphosis deformity, reducing the risk of vertebral body recompression, and reducing long-term pain [17].

Symmetrically distributed bone cement is clinically more effective than laterally distributed bone cement in maintaining biomechanical equilibrium and preventing vertebral fractures and fractures in adjacent segments. The results of finite element analysis performed by Zhang et al. showed that a symmetrical continuous distribution of bone cement over adjacent segments produced about 10% of the stress of the remaining forms of distributed bone cement [18]. Only when the cement distribution crosses the midline and is adequately filled in a symmetrical distribution, the stiffness on both sides increases relatively, thus achieving biomechanical equilibrium [19]. Clinically, after UPKP the bone cement is typically confined to one side of the vertebral body without effectively spreading across the midline (Fig. 3), and the stiffness of the vertebral body on the uncemented side is considerably reduced. The biomechanical imbalance can exacerbate the pressure load on the spine, leading to irreversible effects such as loss of height of the fractured vertebral body, disc degeneration in adjacent segments, and even vertebral fractures in adjacent segments [20]. A biomechanical study by Dai et al. demonstrated that BPKP balanced vertebral stresses, reduced maximum disc stresses, and was more stable than UPKP [21]. Hou et al. conducted a retrospective study and found that symmetrical distribution of bone cement in the vertebral body significantly reduced the incidence of vertebral recompression [22]. Symmetric distribution of bone cement will provide better mechanical support and prevent minor fracture movement, which not only helps to reduce the risk of vertebral recompression but also helps to reduce recompression-induced pain [23]. BPKP offers biomechanical advantages over UPKP in reducing the risk of adjacent vertebral fractures. He et al. classified the distribution of bone cement within the vertebral body into O- and H-type. The authors concluded that the H-type form of cement distribution reduces damage to the surrounding bone by increasing the contact area to prevent injury accumulation in the filling area and impeding vertebral collapse, whereas unbalanced cement distribution subsequently leads to unbalanced mechanical loading within the vertebral body after surgery and may result in accelerated degeneration of adjacent segments [24]. Zhou et al. also found that the incidence of injured vertebral re-fractures and adjacent vertebral fractures was significantly higher in patients with one-sided cement than in the symmetrically distributed type. Furthermore, the more remote the location, the higher the incidence of distant vertebral re-fractures and adjacent vertebral fractures, indicating that asymmetry in cement distribution impacts the long-term outcome of vertebroplasty [25]. Thus, symmetric distribution of bone cement has advantages in restoring spinal stability and reducing postoperative complications such as vertebral recompression, new fractures of adjacent vertebrae, and degenerative disc disease by better restoring the biomechanics of the spine. We believe that symmetrical distribution of bone cement facilitates a tighter connection between bone cement and cancellous bone, increases vertebral strength and stiffness, and reduces the likelihood of vertebral height loss and humpback deformity after PKP. When bone cement is distributed on one side, it cannot maximize the stabilization of the local micro-movement of the fractured vertebral body and destroy the local nerve endings, which may lead to ineffective pain relief. Moreover, owing to the biased load transfer within the vertebral body, the fractured vertebral body is in an unbalanced state with the adjacent vertebral body, and the unevenly distributed biological stresses are transferred to the disc and adjacent vertebral body, which may lead to re-fracture and adjacent vertebral body fracture.

Homogeneous (spongy) dispersion of bone cement provides better clinical pain relief and prevention of vertebral re-fracture than does mass distribution of bone cement. Adequate dispersion of the cement in the fracture line of the injured vertebra provides rapid stabilization and pain relief. Poor dispersion of the cement in the fracture line of the injured vertebrae results in poor pain relief, residual back pain and vertebral fractures. We hypothesize that the spongy dispersion of the bone cement allows for greater contact between the cement and the cancellous bone within the vertebral body, which can adequately fix the fracture fragment, increase spinal stability and reduce micromovement of the trabeculae, which can effectively reduce pain by reducing the irritation of nerve endings within the injured vertebrae. Uneven distribution of bone cement within the vertebral body is a critical risk factor for the development of long-term complications after surgery [23]. As long as bone cement is distributed symmetrically and diffusely within the fractured vertebral body, better rapid pain relief and reduced incidence of distant complications can be obtained [26]. Lin and He et al. both found that the incidence of recompression of the fractured vertebral body was higher when the cement distribution was clump-like and discontinuous, whereas homogeneous distribution of bone cement reduced the incidence of re-fracture of adjacent vertebral bodies [27] [28]. A study by Yu et al. also confirmed that a relatively dispersed distribution of bone cement has better medium- to long-term clinical outcomes than a clumped distribution [29]. However, we also noted that there was significantly more leakage of bone cement with a homogeneous diffuse distribution, indicating that the diffuse form of bone cement is more prone to leakage intraoperatively. AS fractures within the vertebral body are not uniformly and evenly distributed, it is not advisable to push bone cement with sufficient uniform dispersion in the vertebral body to avoid excessive leakage of the bone cement and serious complications.

### Limitations

The present study was a retrospective single-center study with a small number of enrolled cases. Since no adjacent vertebral fractures occurred in the patients during the follow-up time, the effect of puncture access on the incidence of adjacent vertebral fractures is not discussed herein.

Compared with UPKP, BPKP bone cement distribution is more symmetrical and homogeneous, can maintain correction of local scoliosis deformity, maintain biomechanical stability, can relieve local pain and maintain clinical efficacy. BPKP is required for the treatment of patients with AVCF.

## Data Availability

The datasets used and/or analysed during the current study available from the corresponding author on reasonable request.

## List of abbreviations

OVCF: Osteoporotic vertebral compression fracture
PKP: Percutaneous kyphoplasty
AVCF: Asymmetric osteoporotic vertebral compression fractures
VAS: Visual analogue scale
JOA: Japanese Orthopaedic Association
BPKP: Bipedicular Percutaneous Kyphoplasty
UPKP: Unipedicular Percutaneous Kyphoplasty
DVH: Vertebral body height
